# Effects of Nanowire Length and Surface Roughness on the Electrochemical Sensor Properties of Nafion-Free, Vertically Aligned Pt Nanowire Array Electrodes

**DOI:** 10.3390/s150922473

**Published:** 2015-09-04

**Authors:** Zhiyang Li, Calvin Leung, Fan Gao, Zhiyong Gu

**Affiliations:** Department of Chemical Engineering, University of Massachusetts Lowell, One University Ave, Lowell, MA 01854, USA; E-Mails: Li_Zhiyang@student.uml.edu (Z.L.); Calvin_Leung@student.uml.edu (C.L.); Fan_Gao@uml.edu (F.G.)

**Keywords:** Pt nanowire array, electrochemical biosensor, nanowire length, surface roughness, Nafion free, hydrogen peroxide

## Abstract

In this paper, vertically aligned Pt nanowire arrays (PtNWA) with different lengths and surface roughnesses were fabricated and their electrochemical performance toward hydrogen peroxide (H_2_O_2_) detection was studied. The nanowire arrays were synthesized by electroplating Pt in nanopores of anodic aluminum oxide (AAO) template. Different parameters, such as current density and deposition time, were precisely controlled to synthesize nanowires with different surface roughnesses and various lengths from 3 μm to 12 μm. The PtNWA electrodes showed better performance than the conventional electrodes modified by Pt nanowires randomly dispersed on the electrode surface. The results indicate that both the length and surface roughness can affect the sensing performance of vertically aligned Pt nanowire array electrodes. Generally, longer nanowires with rougher surfaces showed better electrochemical sensing performance. The 12 μm rough surface PtNWA presented the largest sensitivity (654 μA·mM^−1^·cm^−2^) among all the nanowires studied, and showed a limit of detection of 2.4 μM. The 12 μm rough surface PtNWA electrode also showed good anti-interference property from chemicals that are typically present in the biological samples such as ascorbic, uric acid, citric acid, and glucose. The sensing performance in real samples (river water) was tested and good recovery was observed. These Nafion-free, vertically aligned Pt nanowires with surface roughness control show great promise as versatile electrochemical sensors and biosensors.

## 1. Introduction

In recent years, there has been increasing demand for sensor design and development for H_2_O_2_ detection, due to the fact that H_2_O_2_ is a very important molecule that is involved in many important fields, such as disease diagnosis [1], environmental protection [2,3], food quality control [4,5], fuel cells [6,7], and the bleach and disinfectant industry [8,9,10,11]. Especially in clinical studies, H_2_O_2_ has been found to play a critical role in a lot of chemical reactions occurring in human body [12,13,14,15]. For example, it is found that an increased level of H_2_O_2_ may facilitate the generation of the most potent hydroxyl radical in the human brain [16]. H_2_O_2_ is one of the final products of most enzymatic reactions, such as glucose oxidation reaction [17,18]. H_2_O_2_ is present in the air exhaled by healthy human subjects [19]; quantities of H_2_O_2_, at concentrations sometimes more than 100 μM, can be detected in freshly voided human urine, even in babies [20]. Some studies have claimed that the level of H_2_O_2_ plays important roles in human blood plasma and blood cells [21]. H_2_O_2_ is a ubiquitous molecule that can be detected in beverages, rain water and waste water [2,3]. Precise detection of H_2_O_2_ can lead to a quantitative measurement of relevant reagents or processes in various applications.

As some of the most widely studied nanomaterials, nanowires have been intensively explored as sensor materials for improved electrocatalytic performance toward H_2_O_2_ reduction, due to their high conductivity, superior stability, and large surface area to volume ratio [22,23,24,25]. Nanowires can be readily synthesized with various materials by using different methods, such as chemical vapor deposition (CVD) [26], the Vapor-Liquid-Solid (VLS) technique [27], surfactant/polymer assisted chemical reduction [28], electroless deposition [29], and electroplating using nanoporous templates [30,31]. Due to their high aspect ratio and unique surface properties, nanowires possess excellent electrochemical and catalytic performance. Among various nanomaterials, platinum (Pt) is one of the most studied noble metals in the sensor and catalyst fields [32,33,34,35,36,37,38,39,40,41,42]. Pt nanowire-based sensors have many applications, especially in the detection of H_2_O_2_, which is attributable to the fact that the decomposition of hydrogen peroxide into water and oxygen can be effectively catalyzed by Pt. In many researches, a nanowire-modified glassy carbon electrode (GCE) has been generally used as the working electrode to detect analytes [33,34,35]. However, directly coating the nanowires onto the electrode surface will result in some disadvantages that compromise the nanowires’ electrocatalytic activity. Since the nanowires are randomly distributed on the two dimensional (2D) working area of the GCE, nanowires tend to form disarrayed and layered films with severe aggregation. Also, since most nanowires are embedded below a top layer of Nafion film, the modified electrode normally results in incomplete contact between the nanowires and the analyte, which leads to material waste and poor electrochemical performance. Furthermore, Nafion is necessary for this modification method to keep the nanowires from dissolution into the solution; however, if bubbles are generated too quickly, some of them may be trapped within the Nafion layer, which may lead to lower electrochemical sensing performance, an even permanent damage to the modified GCE.

To overcome the disadvantages of conventional GCE modification methods, vertically aligned 3D-nanowire array structures have been proposed and reported recently [17,31,36]. In the 3D structure configuration, all nanowires are attached onto a substrate and kept free standing, so the vertical nanowires will not suffer from layered film or aggregation issues. By using the template-assisted electrochemical synthesis of vertical nanowire arrays, nanowires are well-organized based on the distribution of holes on the template, and Nafion is not necessary for nanowire immobilization, which results in better signal quality and sensor durability. Also, vertical aligned nanowires will facilitate the conduction of electrons with a better efficiency, compared to the configuration of nanowires randomly lying parallel to the electrode. In addition, without the presence of Nafion, the vertically aligned structure can also significantly enhance the diffusion of the analyte and products in the solution and adsorption onto the electrode surface. In some researches it has been proved that perpendicularly aligned nanowire arrays can be a significant advancement as sensing devices [31,36,40].

In the 3D nanowire array structure, the length and surface condition of the nanowires are two key factors because they can increase the electroactive working area significantly. However, few studies have been conducted to quantify their influence on sensor performance. In the present work, by controlling the electrodeposition time and current density, we synthesized PtNWAs with different surface roughnesses (smooth and rough) and various lengths (3, 6, and 12 μm), and investigated their electrochemical sensing characteristics through cyclic voltammetric and chronoamperometric measurements. Both the sensitivity and anti-interference properties of the vertically aligned nanowire electrodes were studied. Finally, real water samples were used to test the potential application of these new nanowire-based electrochemical sensors.

## 2. Experimental Section

### 2.1. Apparatus and Reagents

Anodic aluminum oxide (AAO) membrane (Anodisc, 25 mm diameter, 60 μm thickness, 200 nm pore diameter) was purchased from GE Healthcare Life Sciences (Pittsburgh, PA, USA). Pt electroplating solution (Platinum TP RTU, Technic Inc., Cranston, RI, USA) was directly used as purchased. The chemicals for sensing measurements, including hydrogen peroxide (35%), citric acid (99%), D-(+)-glucose (reagent ACS, anhydrous), ascorbic acid sodium salt (99%) and uric acid (99+%), were purchased from Acros Organics (Morris Plains, NJ, USA) and used without further purification. The phosphate buffer solution (PBS) was prepared using sodium phosphate (Acros Organics) and DI water.

A VersaSTAT 3 electrochemical station (Princeton Applied Research, Oak Ridge, TN, USA) was used for electrodeposition, cyclic voltammetry (CV) scanning and chronoamperometry measurement. The morphology and elemental characterization of Pt nanowires were measured by a JSM-7401F field emission scanning electron microscope (FE-SEM) equipped with an energy-dispersive X-ray spectroscopy (EDS) detector (JEOL, Peabody, MA, USA) and Philips EM400T Transmission Electron Microscope (San Francisco, CA, USA).

### 2.2. Synthesis and Fabrication of PtNWA

**Scheme 1:**
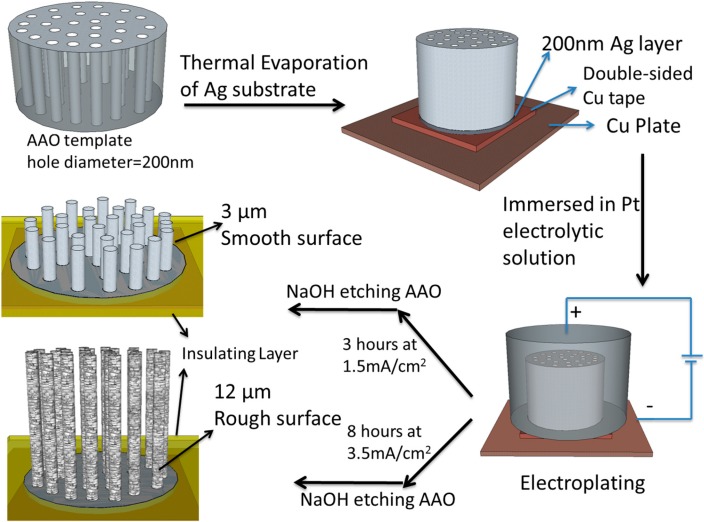
Schematic illustration of the fabrication process for the smooth and rough surface Pt nanowire array (PtNWA).

The PtNWA electrodes were fabricated by electrodeposition in the AAO membrane [41,42,43] as shown in Scheme 1. First, one side of the AAO membrane was coated with a 200 nm thick Ag layer in a CHA 6 Pocket Electron Beam Evaporator (CHA’s Solution™ Process Development System, Fremont, CA, USA) at the rate of 0.1 nm·s^−1^. This Ag layer was conductive and also used as the substrate to support the Pt nanowires that will be electrodeposited. Then, the AAO membrane with the Ag layer was adhered by a piece of double-sided Cu tape (Copper Conducting Tapes, Code 1182, 3M^®^, Maplewood, MN, USA) to a pure Cu plate (1 cm × 5 cm, 0.8 mm thickness), and the other side of the AAO membrane was connected to an O-ring joint with a glass container which was filled with the Pt electrolytic solution. Different current density values (1.5 mA·cm^−2^ for smooth surface nanowires and 3.5 mA·cm^−2^ for rough surface nanowires) and processing times (3 h, 6 h and 12 h for smooth surface nanowires; 2 h, 4 h, and 8 h for rough surface nanowires) were applied in the galvanostatic method to prepare Pt nanowires with different surface roughnesses and various lengths. Electrolytics solutions were refreshed hourly to maintain sufficient Pt ions during the electrodeposition process. After the electrodeposition, the AAO membrane was dissolved in 1 M NaOH solution for 30 min so as to free the vertical nanowires. Finally, the PtNWA was washed three times with DI water and ethanol. To make it a functional electrode, an insulating tape was used to cover the Cu plate and Cu tape to ensure that only the PtNWA electrode could get in contact with the analyte. The Cu plate only played the role of supporting the nanowire array sensor and conducting electrochemical signal.

### 2.3. Electrochemical Measurements

A three-electrode sensing setup was used, including a Pt wire counter electrode (99.9%, Alfa Aesar, Ward Hill, MA, USA), an Ag/AgCl reference electrode stored in 3 M NaCl solution (Bioanalytical Systems, Inc., West Lafayette, IN, USA), and the prepared PtNWA as the working electrode. The electrochemical measurements were carried out in 0.1 M PBS buffer solution (pH = 7.2). For the amperometric measurements, the solution was kept stirring to obtain a uniform concentration of H_2_O_2_. All tests were conducted at room temperature.

## 3. Results and Discussion

### 3.1. Characterization of Pt Nanowires and Array Electrodes

**Figure 1:**
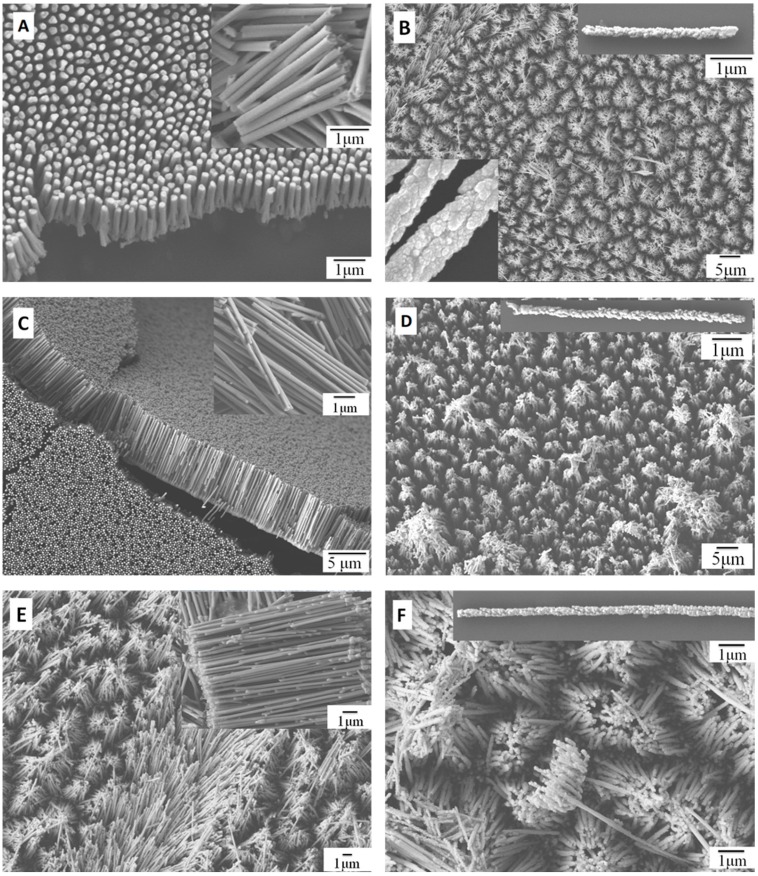
FESEM images of (**A**) 3 μm PtNWA with smooth surface; (**B**) 3 μm PtNWA with rough surface; (**C**) 6 μm PtNWA with smooth surface; (**D**) 6 μm PtNWA with rough surface; (**E**) 12 μm PtNWA with smooth surface; (**F**) 12 μm PtNWA with rough surface.

The structure and morphology of the PtNWAs were examined by SEM as shown in Figure 1. Figure 1A,C,E show the nanowires when the electrodeposition times were 3 h, 6 h and 12 h at a current density of 1.5 mA·cm^−2^. In all three nanowires, the surfaces were smooth and the average lengths of the smooth surface PtNWAs were 3, 6 and 12 μm, respectively (see insets of Figure 1A,C,E). Figure 1B,D,F show the nanowires when the current density was increased to 3.5 mA·cm^−2^. At this higher current density, rough surface textures were observed for all the three nanowires prepared at this condition. In order to obtain nanowires with similar lengths, reduced electrodeposition times were used for surface rough Pt nanowires (2 h, 4 h and 8 h for 3, 6 and 12 μm long nanowires), because a higher current density will lead to faster deposition and thus less deposition times are needed.

The smooth surface Pt nanowires of up to 6 μm maintained a vertical position with uniform distribution on the electrode; however, the longer Pt nanowires (12 μm, Figure 1E) tended to tilt and the nanowires formed clusters close to the top surface. It is believed that when the nanowire length increases, the higher center of gravity makes their position unstable and easy to tilt during the washing and processing stage. The bonding between the nanowires and the substrate is still strong enough to hold the nanowires, however, unable to keep them completely vertical. The rough surface Pt nanowires (Figure 1B,D,F), as shown in our previous researches [41,42], are believed to be due to larger grain sizes formed in the nanowires during the electrodeposition procedure [41]. For instance, Figure 1A,C,E show the smooth surface of the PtNWA prepared at low current density (1.5 mA·cm^−2^) with different deposition times, indicating that small nuclei were formed and these small nuclei packed the whole pore area of the AAO template very well, thus the surface of the nanowires looks smooth and uniform. Figure 1B,D,F show that the surface morphology of PtNWAs that were prepared at high current density (3.5 mA·cm^−2^) is very rough (see insets).

One of the possible reasons of forming a rough surface is that the grain size increases with the increase of the current density, thus the large Pt grains could not uniformly pack the pores of the AAO template during the deposition process. For surface rough Pt nanowires (Figure 1B,D,F), it is believed that the bonding between the nanowires and the substrate was compromised due to the large grain size formed, and thus the nanowires tended to bend and aggregate more easily, resulting in nanowire clusters.

**Figure 2:**
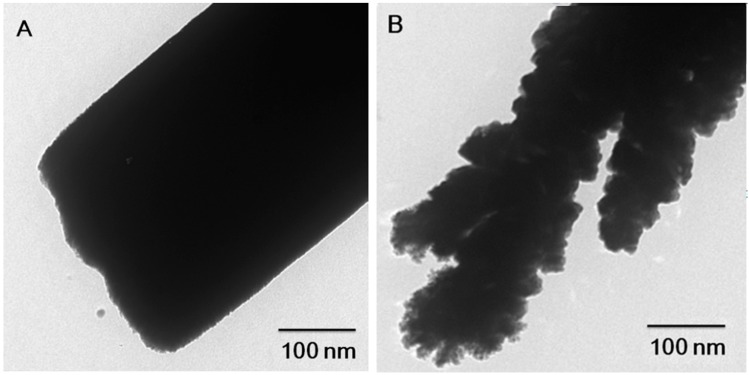
TEM images of (**A**) smooth; and (**B**) rough surface Pt nanowires.

The TEM images of 3 μm long Pt nanowires synthesized at 1.5 mA·cm^−2^ and 3.5 mA·cm^−2^ are shown in Figure 2A,B, Figure 2A shows that the Pt nanowires prepared in lower current density have very smooth and uniform surface conditions, and their internal structure is solid, as expected. Figure 2B presents the rough surface Pt nanowires deposited at high current density, and a number of large grains can be found which are consistent with the SEM images as shown in Figure 1B. As shown in Figure 2B, large grain size can be observed in the surface rough nanowires, which compares well with our previous publications [41,42].

**Figure 3:**
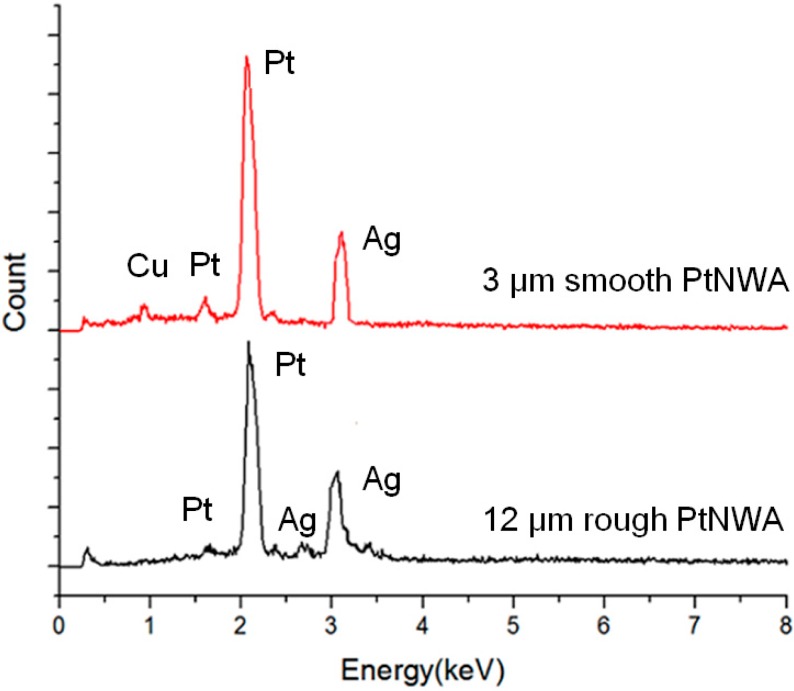
EDS spectra of smooth PtNWA and rough PtNWA.

Figure 3 showed the EDS spectra of both the smooth and rough surface PtNWAs. The spectra are almost identical, indicating that although with different lengths and surface roughnesses, the elements of the Pt nanowires remain the same. The signals of Ag were from the substrate that was holding the nanowire samples. A weak signal of Cu was detected in 3 μm smooth surface PtNWA due to the Cu plate holding the nanowires and the short Pt nanowires for this sample. For the 12 μm long surface rough PtNWA sample, the X-ray was able to detect Ag substrate, however, it was unable to detect the Cu plate (below the Ag layer).

### 3.2. Electrochemical Response of the PtNWA towards H_2_O_2_

**Figure 4:**
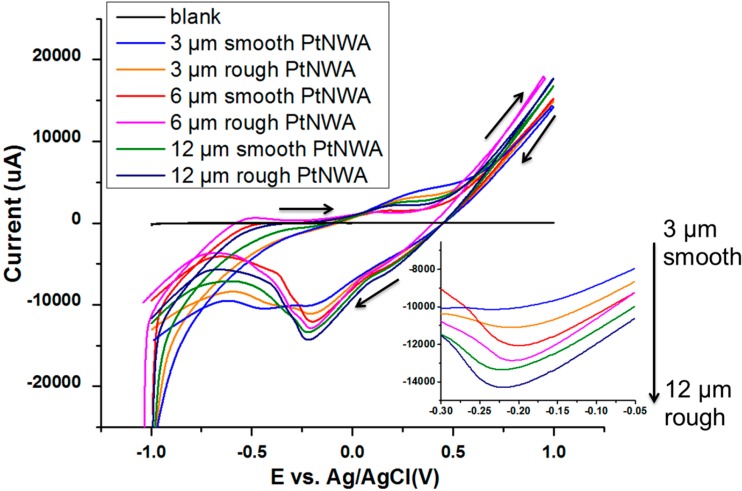
CVs of PtNWA electrodes towards 1 mM H_2_O_2_ with different lengths and surface roughnesses.

The electrocatalytic activity of the PtNWA electrodes toward H_2_O_2_ detection was first evaluated by comparing their cyclic voltammetry (CV) response. Figure 4 shows the steady-state CVs for 3, 6, and 12 μm PtNWAs with smooth and rough surface nanowires in 1 mM H_2_O_2_ PBS solution (pH = 7.2), scanning between −1 V and 1 V potential range, with a scan rate of 0.1 Vs^–1^.

Pt nanowires act as a catalyst for the decomposition of H_2_O_2_. In the presence of PtNWA, electrochemical redox reaction of H_2_O_2_ takes place on the surface of each Pt nanowire. The mechanism of this reaction and the standard electrode potentials relative to the standard hydrogen electrode (SHE) are shown as follows:

(1) $$H_{2}O_{2}\overset{PtNWA}{\to }2H^{+}+\;O_{2}+\;2e^{-},\;E{}^{\circ}\left(V\right)\;=\;-0.68\left(\mathrm{anodic}\right)$$

(2) $$H_{2}O_{2}+\;2H^{+}+2e^{-}\to \;2H_{2}O,\;E{}^{\circ}\left(V\right)\;=\;1.78\left(\mathrm{cathodic}\right)$$

(3) $$2H_{2}O_{2}\to \;2H_{2}O\;+\;O_{2}\left(\mathrm{overall}\right)$$

**Figure 5:**
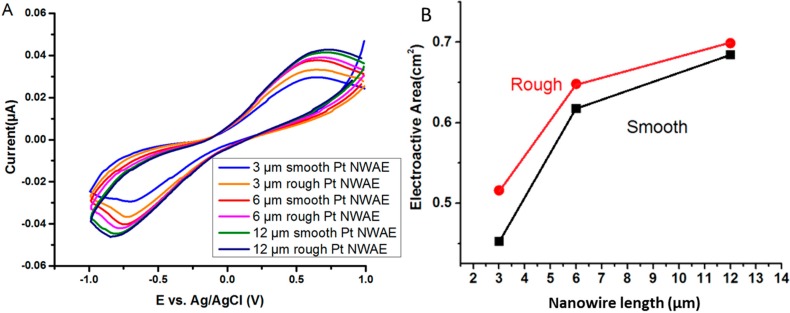
(**A**) CVs of different PtNWAs in 20 mM K_3_Fe(CN)_6_ containing 0.2 mM KCl; the scan rate is 0.1 Vs^−1^; (**B**) The plot of the measured electroactive surface area *vs.* nanowire length of different PtNWAs.

Figure 4 presents the corresponding current-voltage responses of smooth and rough surface PtNWA electrodes with different lengths towards 1 mM H_2_O_2_. The results clearly demonstrate that all PtNWA showed electrochemical response for sensing H_2_O_2_, and PtNWA with different lengths and surface roughnesses have significantly different peak values toward H_2_O_2_ detection. A strong anodic peak potential around −0.2 V can be clearly distinguished on the reverse scans, which means that the PtNWA can catalyze the decomposition of H_2_O_2_ effectively. From 3 μm smooth PtNWA to 12 μm rough PtNWA, the increasing anodic peak potentials proved that the PtNWA has great electrocatalytic property for the redox reaction of small amount of H_2_O_2_. The length and roughness will enhance its electrochemical performance effectively, which should be attributed by increased catalytic surface area and well-ordered nanowire distribution. In addition, the working potential of −0.2 V *vs.* Ag/AgCl was statically applied for the following amperometric detection measurement, in order to avoid possible oxygen reduction and obtain the strongest electrochemical signal. The Randles-Sevcik equation has been widely used in cyclic voltammetry for redox reactions to describe the relationship between the peak current *I_p_* and the various parameters [38,39]. Ferrocene/ferrocenium is often used as a reference redox couple. Figure 5A represents cyclic voltammetry tests of the PtNWAs with different lengths and surface roughnesses in 20 mM K_3_Fe(CN)_6_ containing 0.2 mM KCl with a 0.1 Vs^−1^ scan rate. The obvious oxidation and reduction peaks were observed due to Fe^3+^/Fe^2+^ redox couple at −0.72 V and +0.63 V, respectively. According to the Randles-Sevcik Equation (4):

(4) $$I_{p}=2.69\times 10^{5}AD^{\frac{1}{2}}n^{\frac{3}{2}}{\text{γ}}^{\frac{1}{2}}C$$

the electroactive surface area (A) is a linear function of the peak current of the redox when the diffusion coefficient (D = 6.7 × 10^−6^ cm^2^·s^−1^), number of electrons (*n* = 6), scan rate (γ = 0.1 V·s^−1^), and concentration (C = 2 × 10^−5^ mol·cm^−3^) are constant [39]. Figure 5B shows the relation of the electroactive surface area with surface roughness and nanowire length. It can be seen from Figure 5B that by increasing the nanowire length and surface roughness, the electroactive surface area can be increased effectively and significantly. For instance, the electroactive surface area of 12 μm rough PtNWA is 0.668 cm^2^ and five times larger than the geometric area of the electrode (0.132 cm^2^) [34]. However, the electroactive area of both smooth and rough surface nanowires did not increase linearly with the length of the nanowires. It is believed that as the nanowire length increases, more nanowires are likely to cluster with each other, as shown in Figure 1E,F, resulting in the nonlinear relationship between the nanowire length and electroactive surface area.

### 3.3. Amperometric Measurement

**Figure 6:**
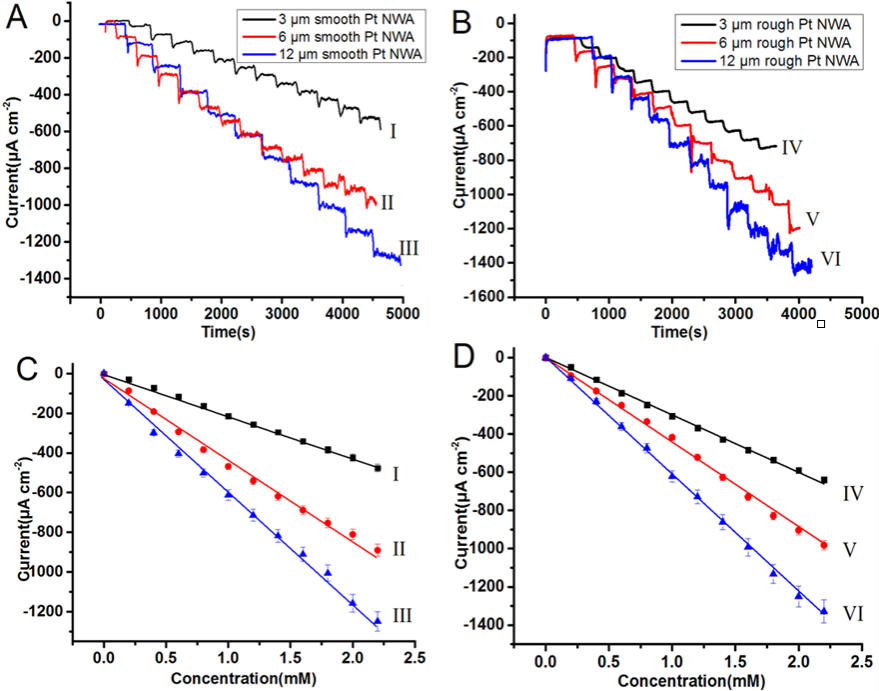
The amperometric response of the following fabricated sensors to the successive addition of 0.2 mM H_2_O_2_ in the 0.1M PBS solution at the potential of −0.2 V *vs.* Ag/AgCl (**A**) 3/6/12 μm smooth PtNWA (I, II, III); (**B**) 3/6/12 μm rough PtNWA (IV, V, VI); (**C**) corresponding plot of 3/6/12 μm smooth PtNWA (I, II, III); (**D**) corresponding plot of 3/6/12 μm rough PtNWA (IV, V, VI).

The amperometric responses of PtNWAs were investigated by a successive addition of H_2_O_2_ into a continuously stirring 0.1 M PBS solution at applied potential of E_app_ = −0.2 V *vs.* Ag/AgCl. Figure 6 shows the typical current-time responses on the successive addition of 0.2 mM H_2_O_2_.

All the sensors responded to the successive addition of 0.2 mM H_2_O_2_ and an immediate feedback signal in current was observed after the injections. With the help of stirring, the steady-state current was quickly achieved within 3 s. All the sensors’ electrochemical signal maintained a good linear relationship with the concentration of H_2_O_2_ until the concentration reached around 3 mM, as shown in Figure 6C,D. When the concentration was high (>2 mM), the 12 μm rough PtNWA (VI) sensor started to show more noisy amperometric responses. The corresponding plots of the average catalytic current against the concentration of H_2_O_2_ were shown in Figure 6C,D as well, with an average correlation coefficient of 0.995. The slopes, which represent the sensitivity of the sensors, could be obtained from the plots.

**Figure 7:**
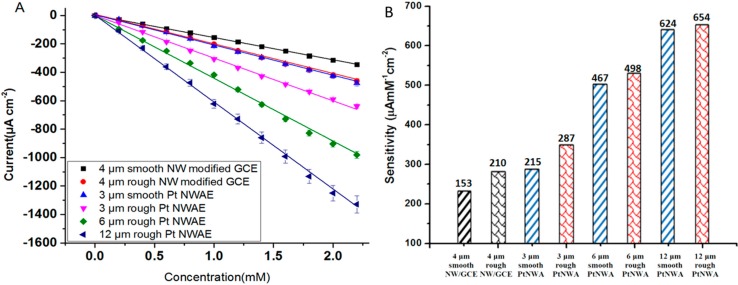
Effect of length and surface roughness on sensitivity (μA·mM^−1^·cm^−2^) of PtNWA.

Figure 7 proves that the sensitivity of PtNWA was increased by increasing the length and surface roughness; one of the possible reasons is the contribution of the significantly increased electroactive area, which is confirmed by the CV scanning results of K_3_Fe(CN)_6_. The sensitivity of 12 μm rough surface PtNWA is the highest due to its largest electroactive surface area (see Figure 7A). The sensitivity of short (3 μm) smooth PtNWA is almost identical to the 4 μm rough PtNW modified GCE, but higher than the 4 μm smooth one [42]. The short (3 μm) rough PtNWA electrode presented a better sensitivity than a 4 μm smooth/rough PtNW modified GCE electrode, which means the well-ordered vertically standing structure can indeed enhance the electrochemical properties. By diluting the analyte to lower concentration (<2.5 mM) the long and rough surface PtNWA sensor could show great sensitivity, low limit of detection, and satisfactory signal quality toward H_2_O_2_ detection. The sensitivity of 12 μm rough surface PtNWA was 3.6 times higher than the 3 μm smooth surface PtNWA. By increasing the nanowire length and its surface roughness, the electrochemical response of PtNWA electrode toward H_2_O_2_ could be enhanced significantly. The comparison of all the sensitivities of the sensors is shown in Figure 7B. The detection limit of 12 μm rough PtNWA was estimated to be 2.4 μM (S/N = 3). The sensitivity of the 12 μm rough PtNWA toward H_2_O_2_ obtained from the plot was 654 μA·mM^−1^·cm^−2^.

**Figure 8:**
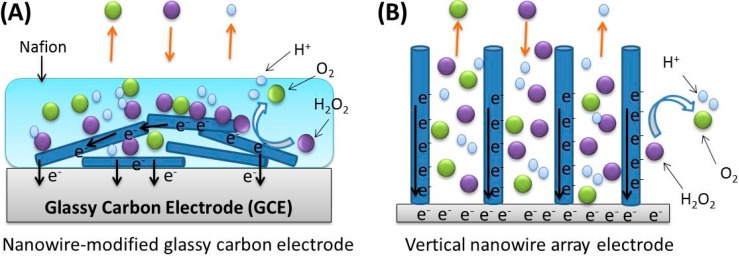
Schematic illustration of the comparison between nanowire-modified GCE *vs.* vertical nanowire array electrode.

From the results above, the vertically aligned Pt nanowire array electrodes showed better performance compared to the conventional nanowire-modified GCE electrodes. In the traditional nanowire modified GCE as shown in Figure 8A, the diffusion of molecules are compromised by the Nafion layer and the randomly layered nanowires, and the electrochemical signal cannot be efficiently conducted either. However, for the vertical nanowire array structure, the analyte (H_2_O_2_) and product molecules can diffuse more freely in the solution and adsorb onto the surface of vertically aligned nanowire electrode. Also, the electrochemical signal can be directly transferred, as shown in Figure 8B, which contributed to better sensing performance.

The overall performance of the 12 μm rough surface PtNWA is compared with different Pt-based sensors in the literature, as shown in Table 1.

**Table 1:** Comparison of electrochemical sensing properties towards H_2_O_2_ detection for various Pt-based nanostructure electrodes.

Electrode	E_app_ (mV)	Sensing Performance			Reference
		LOD ^a^ (μM)	Sensitivity (μA·mM^−1^·cm^−2^)	LDR ^b^ (mM)	
PtNP-CNT array	-	1.5	140	~25	[39]
Smooth Pt NW/GCE	−480 ^c^	47	130	~35	[42]
Rough Pt NW/GCE		20	170	~33	
PtNP/NAE	-	1.0	194.6	0.020–20	[44]
HRP/Pt nanowire	−100 ^c^	-	345	~2.5	[45]
Marcroporous Au/nPts	700^c^	50	264	~0	[46]
GC/RGO/PB/PTBO	200 ^c^	1.5	420	0.005~0.6	[47]
Pt NEA	0	0.06	540	0.0001–60	[38]
12 μm rough PtNWA	−200 ^c^	2.85	654	0.01~4	**This work**

^a^ Limit of detection; ^b^ Linear dynamic range; ^c^ Potential *vs.* Ag/AgCl.

Compared with the conventional GCE modification method [42], the PtNWA successfully lowered the LOD by ten times, and increased the sensitivity by five times. It can be seen that the prepared 12 μm rough surface PtNWA sensor shows an excellent performance in terms of sensitivity and limit of detection (LOD). The 12 μm rough PtNWA sensor exhibited the highest sensitivity, among all the sensors shown in the table. For the limit of detection, the lowest value (0.06 μM) was observed for an enzyme based sensor; however, for non-enzymatic sensors, our value (2.85 μM) is a little higher than PtNP-CNT array structure [39] but comparable. The LDR of the PtNWA sensor was not as wide as some of the reported values, but it is acceptable. This can be explained by the fact that due to the highly ordered structure and uniform distribution, each vertical nanowire can catalyze the decomposition of H_2_O_2_ and contribute to the electrochemical performance. Also, because the Pt nanowires are attached to the substrate directly, electrochemical signals can be effectively transferred and collected by the analysis system. By increasing the length and surface roughness, the enlarged electroactive surface area can enhance the sensor electrocatalytic ability, leading to higher sensitivity and lower detection limit.

### 3.4. Selectivity Measurement

**Figure 9:**
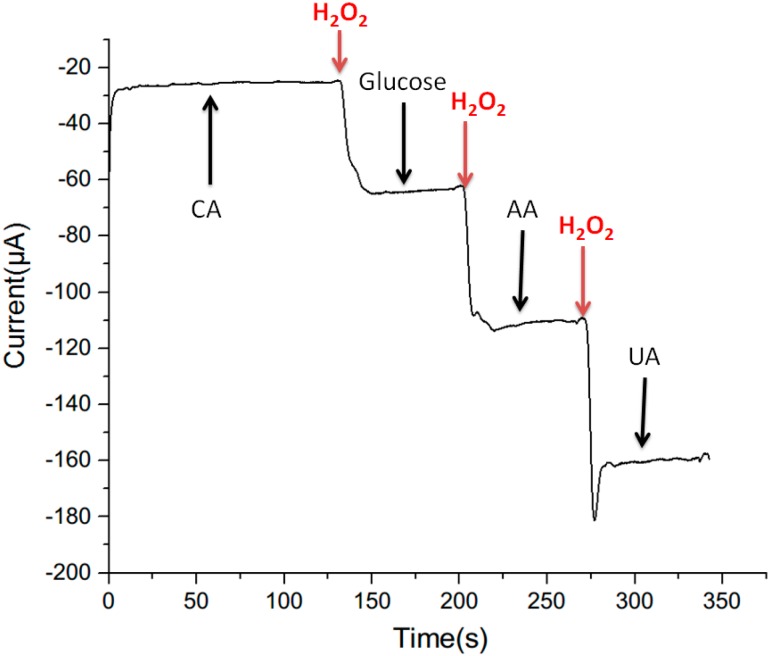
Selectivity measurement of 12 μm rough surface PtNWA with the addition of 0.1 mM CA, H_2_O_2_, glucose, AA, and UA.

The anti-interference ability of all the PtNWA sensors was investigated by adding 0.1 mM of citric acid (CA), glucose, ascorbic acid (AA) and uric acid (UA), sequentially into the solution and the corresponding sensor responses of 12 μm PtNWA with a rough surface are shown in Figure 9. The results demonstrate that the responses from CA, glucose, AA and UA are negligible compared to that was observed for 0.1 mM H_2_O_2_, indicating a high selectivity and good anti-interference properties of long rough structured PtNWA sensor.

### 3.5. Detection of H_2_O_2_ in Real Water Samples

**Figure 10:**
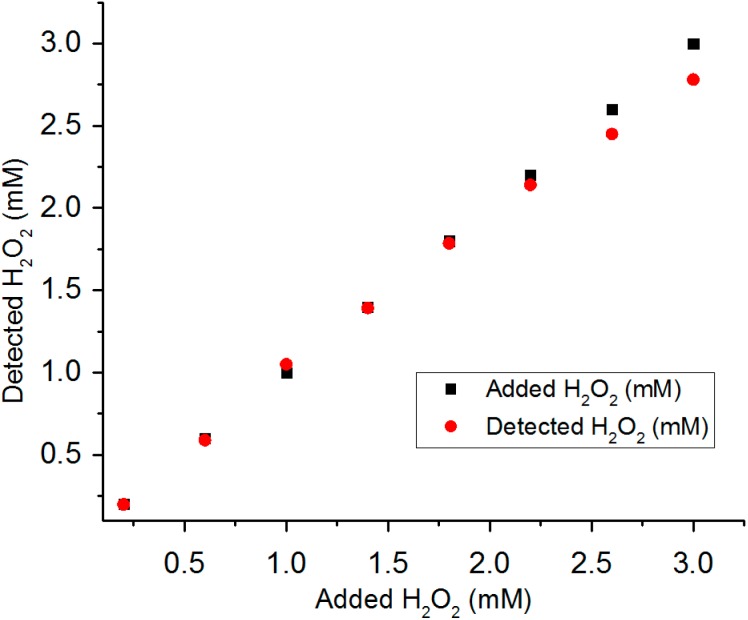
Recovery test of 12 μm rough surface PtNWA toward H_2_O_2_ in real river water sample.

A real water sample was collected from Merrimack River (Lowell, MA, USA) and was directly used without any further purification. A set of H_2_O_2_ solutions with known concentrations was prepared by adding H_2_O_2_ solutions to the real river water sample. The 12 μm rough surface PtNWA was used to detect the H_2_O_2_ and quantify its concentration in the real water sample. The recovery performance of the PtNWA sample was shown in Figure 10. In low H_2_O_2_ concentration range (0.1 mM to 2 mM), the 12 μm rough PtNWA sensor showed excellent H_2_O_2_ sensing capability within the real water sample and demonstrated a recovery of >98.5%. In higher concentration range from 2.5 mM to 3 mM, the recovery of this sensor slightly dropped; however, it could still show satisfactory H_2_O_2_ detection ability with 93.5% recovery.

## 4. Conclusions

A one step electrodeposition method was applied to the synthesis of vertically aligned Pt nanowire arrays on an AAO template. By controlling the current density and deposition time, Pt nanowires with different surface roughnesses were successfully prepared in the length range of 3–12 μm. The vertical Pt nanowires are free standing and well organized, and each nanowire is directly connected to the Ag substrate for the best electrical conductivity. The Nafion-free design of the nanowire array electrode avoids the trapping of bubbles and facilitates the diffusion of analytes, which leads to better sensor performance and durability. It is found that the nanowire length and surface roughness can enhance the nanowire array’s electrochemical properties, which is contributed by the significantly increased active surface area. According to the experimental results, the longest and roughest PtNWA can provide 3.6 times higher sensitivity (654 μA·mM^−1^·cm^−2^) than the short and smooth one (223 μA·mM^−1^·cm^−2^). In addition, this long and rough PtNWA electrode has quick response and great selectivity toward H_2_O_2_ detection and good anti-interference ability with glucose, uric acid, citric acid, and ascorbic acid. The recovery of a real river water sample from the PtNWA is satisfactory. The proposed rough surface Pt nanowire array electrode can serve as a new platform for electrochemical and biomolecular detection.
